# When “Aging” meets “Intelligence”: smart health cognition and intentions of older adults in rural Western China

**DOI:** 10.3389/fpsyt.2024.1493376

**Published:** 2025-06-19

**Authors:** Xiao-Yu Li, Jun Li, Ning-Li Zhu, Lei Luo, Si-Yu Zhang, Wen-Wen Cheng, Jia-Xue Li, Chen Yu, Song-He Lu, Liang Zhu

**Affiliations:** ^1^ School of Preventive Medicine, The Fourth Military Medical University, Xi’an, Shaanxi, China; ^2^ The Shaanxi Provincial Key Laboratory of Environmental Health Hazard Assessment and Protection, Xi’an, Shaanxi, China; ^3^ The Ministry of Education Key Lab of Hazard Assessment and Control in Special Operational Environment, Xi’an, Shaanxi, China; ^4^ Department of Burns and Cutaneous Surgery, The First Affiliated Hospital of the Fourth Military Medical University, Xi’an, Shaanxi, China; ^5^ Scientific Research Department, The Fourth Military Medical University, Xi’an, Shaanxi, China

**Keywords:** smart health, population aging, rural older adults, cognition, behavioral intentions

## Abstract

**Background:**

Population aging is occurring at an unprecedented pace, rendering “healthy aging” a critical issue of global importance. China is at the forefront of this demographic transformation and faces substantial challenges, especially in its western rural regions. In this context, smart health technology, propelled by advancements in medical care and information technology, emerges as a vital strategy to address the challenges associated with aging. Despite smart technologies’ promising potential, the cognitive and behavioral intentions of older adults living in underdeveloped areas remain poorly understood.

**Methods:**

This study focuses on individuals aged 60 years and older residing in the rural regions of Western China. A total of 311 comprehensive datasets were collected using a questionnaire. These datasets encompass the fundamental cognitive understanding, usage requirements, and barriers faced by rural older adults regarding various aspects of their lives. The study aims to explore the attitudes, expectations, and personal perspectives of elderly rural residents towards smart health technology, as well as their views on its adoption. Finally, drawing on the literature, we constructed a structural equation model to analyze the primary factors influencing the cognitive and behavioral intentions of rural older adults toward smart health.

**Results:**

The survey results indicate significant progress in promoting smart health initiatives within the rural areas of western China. While the older adult population demonstrates a basic understanding of smart health, particularly concerning wearable devices such as blood pressure monitors and oximeters, this understanding also underscores the urgent need for improved health management and enhanced quality of life among rural older adults. As their comprehension of smart health deepens, a majority of older respondents recognize its potential to benefit personal health management, fulfill their daily needs, and highlight its importance for rural development. In terms of cognitive pathways toward adopting smart health, older individuals prefer recommendations from their children or spouses, as well as guidance from healthcare professionals such as doctors or nurses. Overall, older residents in rural western China exhibited a strong willingness to embrace smart health practices. After experiencing smart health technology, they were inclined to adopt it as their primary method of health management and recommend it to others, thereby presenting promising prospects for future smart health promotion in underdeveloped regions.

**Conclusion:**

Smart health has immense potential to enhance the quality of life of older adults, driven by a genuine and pressing demand expressed by rural older adults and a strong behavioral intention toward adoption. Despite facing challenges, such as limited cognitive diversity in smart health, economic constraints, technical usability barriers, and lack of social and familial support, future initiatives must prioritize the actual needs of older adults in rural areas. This requires effective communication strategies and service models tailored to the specific conditions of underdeveloped regions in order to expand and deepen the use of smart health applications. Moreover, concerted efforts by governments, enterprises, and all sectors of society are essential to deliver more accessible and cost-effective health management and services to older adults. Ultimately, these efforts will contribute to enhancing the overall well-being and quality of life of older adults.

## 1 Introduction

In October 2022, the World Health Organization’s (WHO) “Aging and Health” report proposed that by 2050, 80% of older adults will come from low- and middle-income countries (1). At the same time, the “World Social Report 2023” of the United Nations Economic and Social Council reported the number of older people in developing countries will increase the most and rapidly, and Asia will become the region with the largest older population (2). In this context, it is crucial to anticipate aging trends and adjust public health policies to support healthy aging in low- and middle-income countries and regions (3).

Healthy aging has emerged as a significant research focus, with many asserting that rural older adults encounter numerous challenges in health management. Firstly, concerning self-management, some argue that rural older adults exhibit poor self-management behaviors, lack health management awareness, and maintain negative attitudes towards self-care (4). Furthermore, when faced with illness, rural older adults often adhere to their daily routines and prefer self-treatment or selectively follow medical advice rather than relying on scientifically based clinical solutions (5). These perspectives highlight critical issues in the health management of rural older adults, including inadequate health awareness, reliance on traditional and self-treatment methods, and the unequal distribution of medical resources and services in rural areas. Consequently, improving health management for rural older adults necessitate a comprehensive approach that considers multiple factors and develops targeted strategies.

Secondly, regarding mental health, feelings of loneliness and a sense of loss are prevalent among rural older adults (6, 7), especially among left-behind elderly individuals, who exhibit higher rates of depressive symptoms than the general elderly population (8).

Thirdly, rural older adults often experience prolonged periods without family and social support. Their adult children, engaged in long-term work away from home, cannot provide timely material, emotional, and practical assistance, negatively impacting overall life satisfaction (9, 10).

For older adults in rural western China, health management poses even greater challenges. Research indicates that the healthy life expectancy for individuals aged 60 and above in rural western areas is lower, and their basic physical condition is generally poorer. Economic constraints render medical resources in these regions scarce, complicating access to quality medical services (11). Additionally, the fragile living environment in rural western areas, marked by significant climate fluctuations, including extreme temperatures and humidity, adversely affects the health of older adults (12, 13). Therefore, enhancing health management for rural older adults in western China is a critical issue in promoting healthy aging and improving their quality of life.

Simultaneously, smart health offers new opportunities. Smart health transcends the temporal and spatial limitations of traditional elderly care models, integrating all service participants—such as government, community, and medical institutions—through modern technology. Smart health technology allows for the real-time monitoring of health data, early detection of health risks, and remote delivery of health services through advanced technologies such as the Internet of Things (14), big data (15), and cloud computing (16). These capabilities facilitate convenient and efficient health management services for older adults, enhancing care and support. Smart devices, such as bracelets and blood pressure monitors, enable real-time monitoring of physical indicators, improving health awareness and self-management. This ease of access is particularly beneficial for older adults living alone in rural western areas (17). In addition, the application of big data, the Internet of Things, and artificial intelligence technologies in health management allows rural older adults in western China to use smart health services, including daily life support, medical care, leisure and entertainment, and emotional comfort (18).

However, significant challenges hinder the adoption and effective utilization of smart health technologies, particularly for older adults with limitations in information technology and cognitive abilities. Can they genuinely accept and integrate these technologies into their daily lives? At the intersection of “aging” and “intelligence,” what factors influence older adults’ acceptance and use of smart health technologies? In rural areas, can implementing smart health technologies significantly enhance healthcare services and overall well-being for older adults? Unfortunately, existing research has seldom addressed these critical issues.

This study investigated the awareness and behavioral intentions of smart health technologies among rural older adults in Western China, providing theoretical support and practical insights for promoting and implementing smart health initiatives in less developed areas. The research results not only contribute to enhancing and broadening the theoretical framework and application of smart technologies in older adults’ health management but also offer valuable references for decision-makers, healthcare providers, and technology developers. The goal of these efforts is to elevate the level of health management for older adults in less developed regions.

## 2 Methods

### 2.1 Sample and sampling

This study is based on a questionnaire survey conducted through the data platform Credamo from May to June 2024. Given the lower education levels among rural older adults in Western China and their limited usage rates of smart devices, we conducted the survey with the assistance of the older adults’ children as proxy respondents. The questionnaire was distributed in two stages. In the first stage, we distributed 1,000 questionnaires for a preliminary survey, including screening questions such as, “Are your parents aged 60 years or older?” and “Do your parents have long-term residency in rural areas of the West?” In the second stage, we collected questionnaires from children of elderly individuals aged 60 or older with long-term residency in rural areas of the West, resulting in 340 valid responses. Statistical analysis revealed that the samples were evenly distributed among the rural areas of 12 western provinces in China, ensuring randomness and avoiding sample selection bias. To promote broad and representative participation, we randomly allocated survey links before distributing the questionnaires and implemented measures such as IP address restrictions, device identification codes, and account login verification to prevent multiple participations by the same respondent. Ultimately, we collected 311 valid questionnaires after excluding individuals who did not pass the attention test. All participants provided informed consent before the survey.

### 2.2 Study procedures

This study employed a hybrid methodology that integrates questionnaire surveys and structural equation modeling (SEM). The questionnaire design adhered to scientific and systematic principles to ensure a comprehensive and accurate reflection of older adults’ cognition and behavioral intentions toward smart health technologies.

The survey focused on the elderly population aged 60 years and older in rural areas of Western China. Detailed data on the cognition, usage needs, and perceived barriers to smart health technologies among rural elderly individuals were collected through questionnaires administered via online surveys, with the children of the elderly individuals responding on their behalf.

### 2.3 Instruments and measurements

The research instruments included structured questionnaire and empirical analysis. Through theoretical analysis, trial runs, and iterative refinements, aligned with the actual circumstances of older adults in rural Western China and the development status of smart health technologies, three distinct sections were finalized for the questionnaire: “Basic personal characteristics,” “Smart health cognition (SHC),” and “Smart health intention (SHI).” These sections were designed to collect the participants’ demographic information, assess their understanding of smart health technologies, and gauge their willingness to adopt these technologies. A Likert scale was employed to quantify the participants’ attitudes, perceptions, and behavioral intentions toward smart health technologies. We initially analyzed the participants’ demographic characteristics, including their gender, age, marital status, education level, living arrangements, monthly family income, and other basic personal traits, to gain a comprehensive understanding of the sample population. Descriptive statistical methods were employed to summarize these characteristics, thereby delineating the demographic profiles of the rural older adults (**Table 1**).

**Table 1:** Sociodemographic characteristics of the respondents.

Characteristics	Respondents(N=311)	Ratio(%)
Gender		
Male	143	46
Female	168	54
Age,years; mean±SD	70.15±7.13	
Age		
60–69 years old	161	51.8
70–79 years old	111	35.7
80 years old and older	39	12.5
Marital status		
Married	245	78.8
Widowed	60	19.3
Divorced	6	1.9
Living status		
Living alone	33	10.6
Living with children	124	39.9
Living with spouse	149	47.9
Living in nursing homes	4	1.3
Living with others	1	0.3
Education level		
No education	11	3.5
Primary school and below	110	35.4
Middle school	111	35.7
High school and above	79	25.4
Monthly household income		
No income	38	12.2
<1000 CNY	50	16.1
1000–1999 CNY	49	15.8
2000–2999 CNY	67	21.5
3000–3999 CNY	55	17.7
>4000 CNY	52	16.7

We employed SEM as the primary analytical to comprehensively explore the complex relationships between the factors that influence older adults’ cognition and behavioral intentions toward smart health technologies. SEM integrates factor, regression, and path analyses, making it well suited for examining the influence of latent variables on observable ones, as well as the intricate interactions between these variables (19, 20). We used a SEM analysis to uncover the internal relationships and influencing factors between rural older adults’ cognition of smart health technologies and their behavioral intentions.

#### 2.3.1 Demographic characteristics

In studies concerning healthy aging, socio-demographic factors, such as gender, age, psychological state, economic status, and health-related behaviors, are commonly identified as key influencers on older adults’ health, with demonstrated correlations to their behaviors (21). This study examined the relationship between smart health cognition and intentions among older adults in rural areas. We considered gender, age, marital status, residential location, education level, monthly household income, and other variables as demographic characteristics within the sample structure.

#### 2.3.2 Smart health cognition

**Figure 1:**
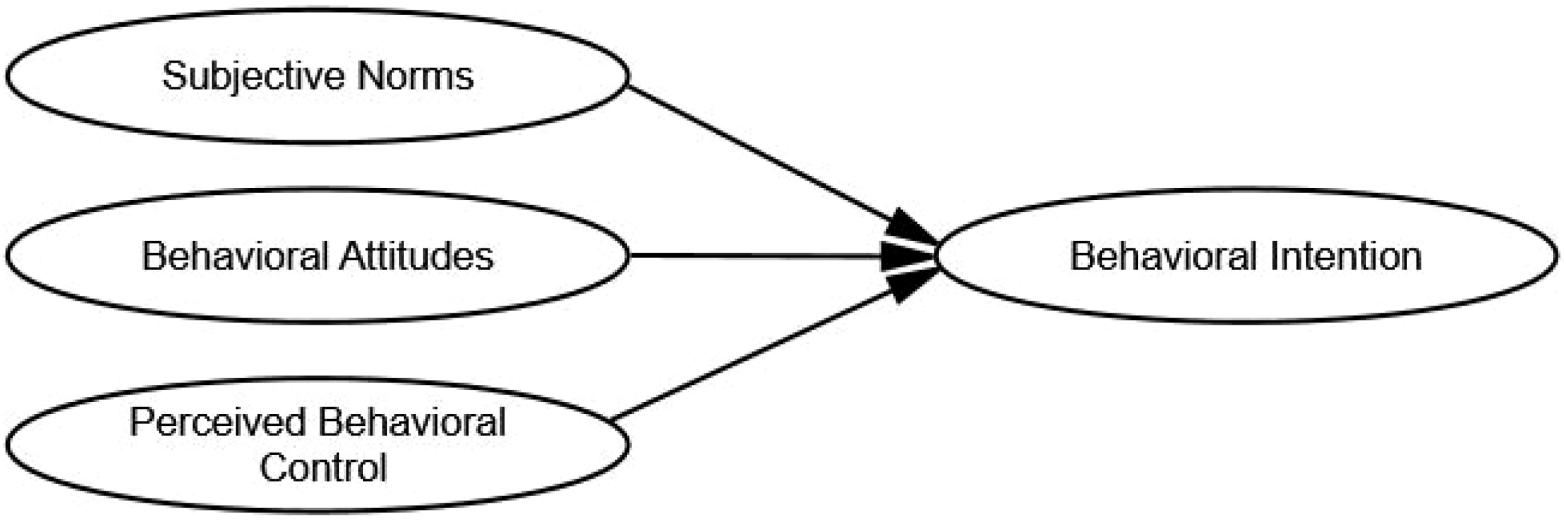
Model of the impact of smart health cognition on behavioral intention.

The theory of planned behavior (TPB) that was first proposed by Ajzen and Fishbein, posits that behavioral intention is a key predictor and explanatory factor of individual behavior (22). According to TPB, the stronger the behavioral intention, the greater the likelihood of performing the behavior in question. It is influenced by three primary factors: behavioral attitudes (BA), subjective norms (SN), and perceived behavioral control (PBC). In this study, we deconstructed SHC in older adults living in rural areas of Western China into BA, SN, and PBC. In this context, SHC represents the psychological expectations or subjective desires of rural older adults regarding their acceptance and adoption of smart health technologies. These factors significantly influence ultimate behavioral intention (BI), specifically SHI (**Figure 1**).

BA represents an individual’s evaluation of a particular behavior, encompassing both value judgments (whether the behavior is good or bad) and outcome evaluations (perceived benefits or disadvantages associated with the behavior) (23). For rural older adults, BA toward smart health technology refers to their assessment of their emotional inclination toward such technology. When designing BA items, we considered rural older adults’ preferences or evaluations of smart health technology, including its alignment with their actual health needs, compatibility with social and economic development, fulfillment of personal health expectations, and the anticipated functions and effects of smart health technologies.

SN refers to the perceived social pressure that individuals experience to either engage in or refrain from specific behaviors and stems primarily from significant others or groups with whom they have close social ties (24). In addition to personal relationships, professionals, such as doctors and nurses, hold authoritative positions and provide crucial medical information and health guidance. Therefore, when designing the SN components, we also considered the support older adults received from family members, relatives, friends, neighbors, doctors, and nurses in the use of smart health technologies.

PBC refers to an individual’s perception of the ease or difficulty associated with performing a specific behavior. This perception is primarily influenced by factors that facilitate or impede the behavior, such as economic circumstances, available resources, and personal capabilities. For older adults living in rural areas in Western China, several key factors influence their PBC toward smart health technologies. These factors include understanding the technical aspects of smart health, the economic environment of their families, their health status, and their overall perception of smart health technologies. When designing components related to PBC, we took into consideration rural older adults’ abilities, resources, and conditions that may impact their ability to use smart health technologies, such as their capacity to learn, economic conditions, and availability of time for usage.

#### 2.3.3 Smart health intention

A primary tenet of TPB is that a positive attitude toward a behavior, strong social norms supporting the behavior, and high perceived behavioral control over performing the behavior produce stronger behavioral intentions. It is crucial to acknowledge that an individual’s cognitive level determines their readiness to act, thereby influencing their behavioral decision-making process (25). Therefore, the intention of rural older adults to participate in smart health behaviors refers to their preparatory state for utilizing smart health technologies, as well as their subjective likelihood of desiring to engage in intelligent health behaviors. The design of the smart health intention measure considers the direct intention of rural older individuals to utilize smart health technologies, their intention to engage in health management, as well as their intention to recommend smart health after its use. All the items and options for SHC and SHI can be found in **Table 2**.

**Table 2:** Results of the variable validity and reliability analysis.

Variable	Measurement dimension	Code	Item	Standardized factor loading	Cronbach’s alpha coefficient	Options
Smart health cognition (SHC)	Subjective norms	SN1	My children or spouse recommend that I use smart health technology	0.654	0.641	1 Strongly disagree 2 Disagree 3 Neutral 4 Agree 5 Strongly agree
		SN2	My relatives or neighbors recommend that I use smart health technology	0.633		
		SN3	My phone recommends that I use smart health technology	0.691		
		SN4	My doctor or nurse recommends that I use smart health technology	0.659		
	Perceived behavioral control	PBC1	I have the ability to learn to use smart health technology	0.651	0.605	
		PBC2	My economic conditions allow me to use smart health technology	0.669		
		PBC3	I have enough time to use smart health technology	0.632		
	Behavior Attitude	BA1	I am very familiar with smart health technology	0.693	0.832	
		BA2	I believe that smart health technology is beneficial for my health management	0.783		
		BA3	I believe that smart health technology meets my life needs	0.809		
		BA4	I believe it is necessary to promote smart health technology in rural areas	0.673		
Smart health intention (SHI)	Behavior Intention	BI1	I am willing to use smart health	0.817	0.768	
		BI2	I am willing to use smart health as my primary health management tool	0.749		
		BI3	If I use smart health, I will recommend it to people around me	0.694		

#### 2.3.4 Reliability and validity analysis

To ensure effective and reliable research outcomes, we used the social science statistical software package SPSS for Windows, version 22.0 (SPSS Inc., Chicago, Illinois, USA) to assess the reliability of the observable variables before conducting the exploratory factor analysis. The results indicated a Cronbach’s alpha coefficient of 0.861 for the overall questionnaire, surpassing the standard threshold of 0.6, which suggests good internal consistency. Furthermore, the standardized factor loadings of all the observed variables ranged from 0.632 to 0.817, exceeding the standard threshold of 0.5. This suggests that the questionnaire structure demonstrated good validity. In terms of the exploratory factor analysis, the Kaiser-Meyer-Olkin (KMO) measure for the sample population was 0.888, with a significance level of Bartlett’s test of sphericity of p < 0.05 which suggests that the sample is suitable for factor analysis (**Table 2**).

#### 2.3.5 Structural equation modeling

**Figure 2:**
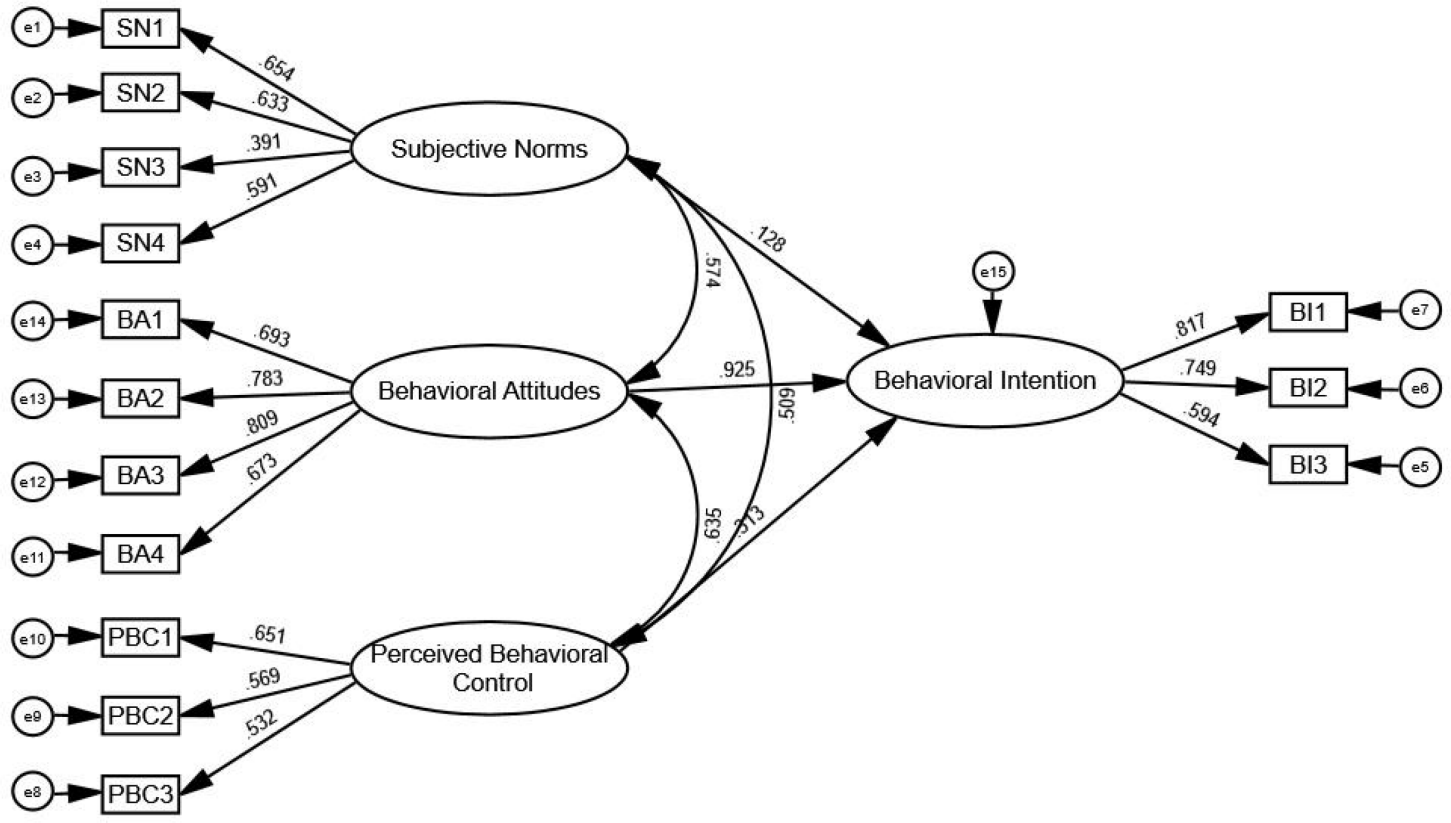
Structural equation modeling of smart health intention.

In order to further calculate the loading coefficient between latent variables and observable variables. We used Amos statistical software (version 20.0) was used to construct and evaluate the SEM, specifically to analyze the factors influencing smart health intentions among rural older adults. **Figure 2** illustrates the SEM of SHI using Amos 20.0.

## 3 Results

### 3.1 Demographic characteristics

The majority of participants were women (54%), with an average age of 70.1 years. Most resided with their spouses or children (88%), and a significant proportion were married (79%). A considerable number had lower levels of education, with 75% having completed secondary school or less. Most reported low monthly family incomes, with 65% earning below 3,000 CNY or having no income.

### 3.2 Smart health cognition

Our survey of the SHC of older adults in rural areas showed that more than half of the participants understood the concept of smart health (58%), indicating a certain level of awareness among rural older adults. Wearable devices, such as blood pressure monitors, oximeters, and health-monitoring instruments, received the highest level of recognition, followed by telemedicine services and mobile health applications.

For older adults who have used or are familiar with health-monitoring equipment, smart health technologies offer real-time reflection of physical indicators such as heart rate, blood pressure, and blood glucose levels. This capability enables them to promptly identify health issues, enhancing their attentiveness to health status and improving health awareness and literacy, particularly in disease prevention and chronic disease management. Consequently, smart health is widely perceived as beneficial for older adults’ health management (83%).

The majority of the rural older adults in our survey also said smart health technology meets their daily life needs (69%). Specifically, the convenience afforded by these technologies allows them to monitor their health status from home, reducing the need for frequent visits to local or higher-level hospitals. This independence fosters greater participation in health management among older adults, leading to enhanced effectiveness in self-monitoring and adjustments.

**Figure 3:**
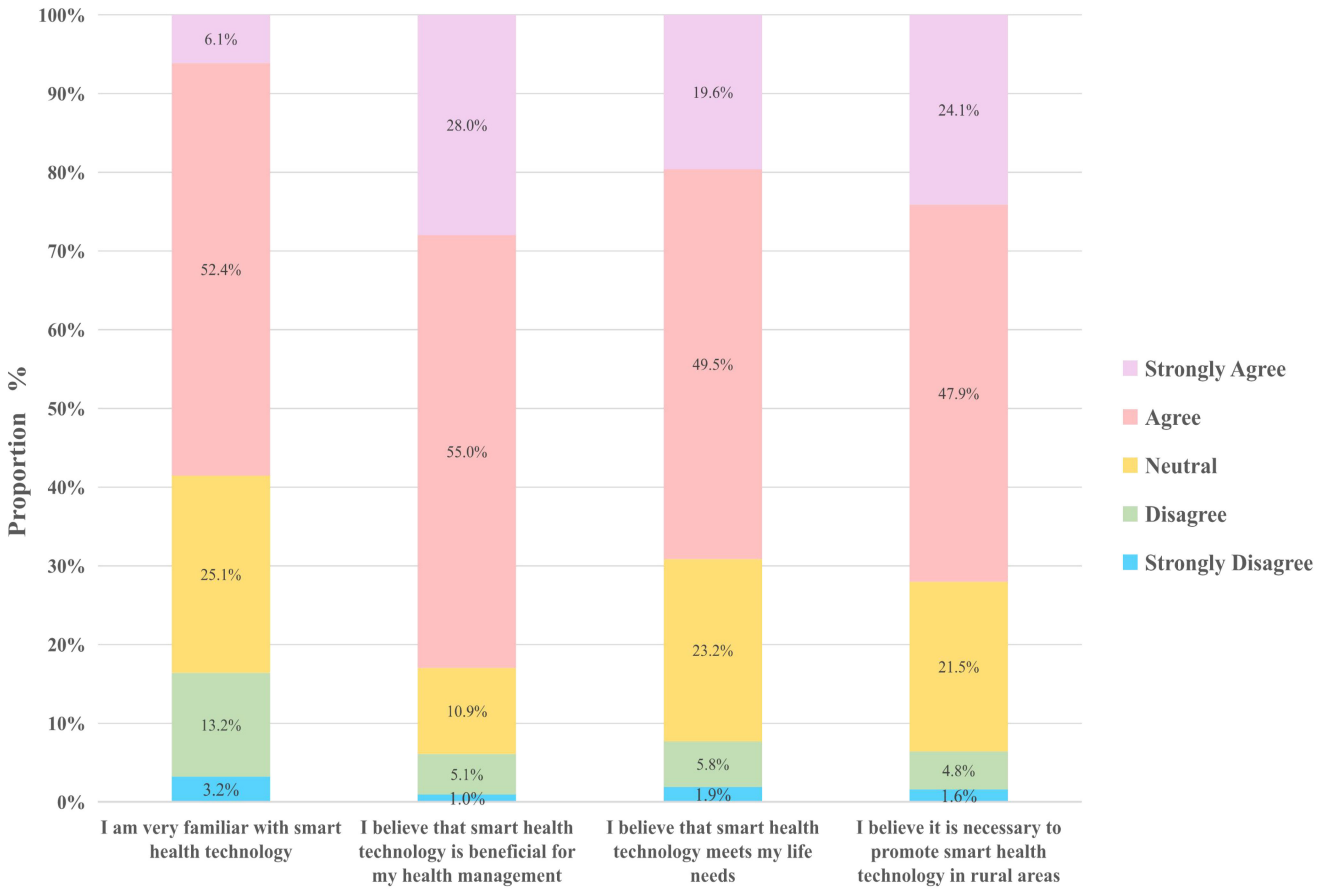
Smart health behavioral attitudes.

Additionally, 72% of the rural older adults in our survey confirmed the necessity of promoting smart health technologies in rural areas. This finding underscores the potential for the future promotion of smart health services in rural and underdeveloped areas, reflecting the urgent demand among older populations for improved health management and medical services (**Figure 3**).

A survey on the SN of smart health among rural older individuals found that the primary source of cognitive influence regarding smart health was children or spouses (88%), followed by recommendations from doctors or nurses (79%). This trend can be attributed to close family relationships typical of rural areas and the authoritative influence of healthcare professionals.

Compared to the recommendations from nonprofessionals, such as relatives, friends, or neighbors (49%), doctors and nurses can better align smart health technologies or services with the specific health conditions and living needs of older adults. This ensures that older adults perceive the practicality and relevance of smart health solutions.

**Figure 4:**
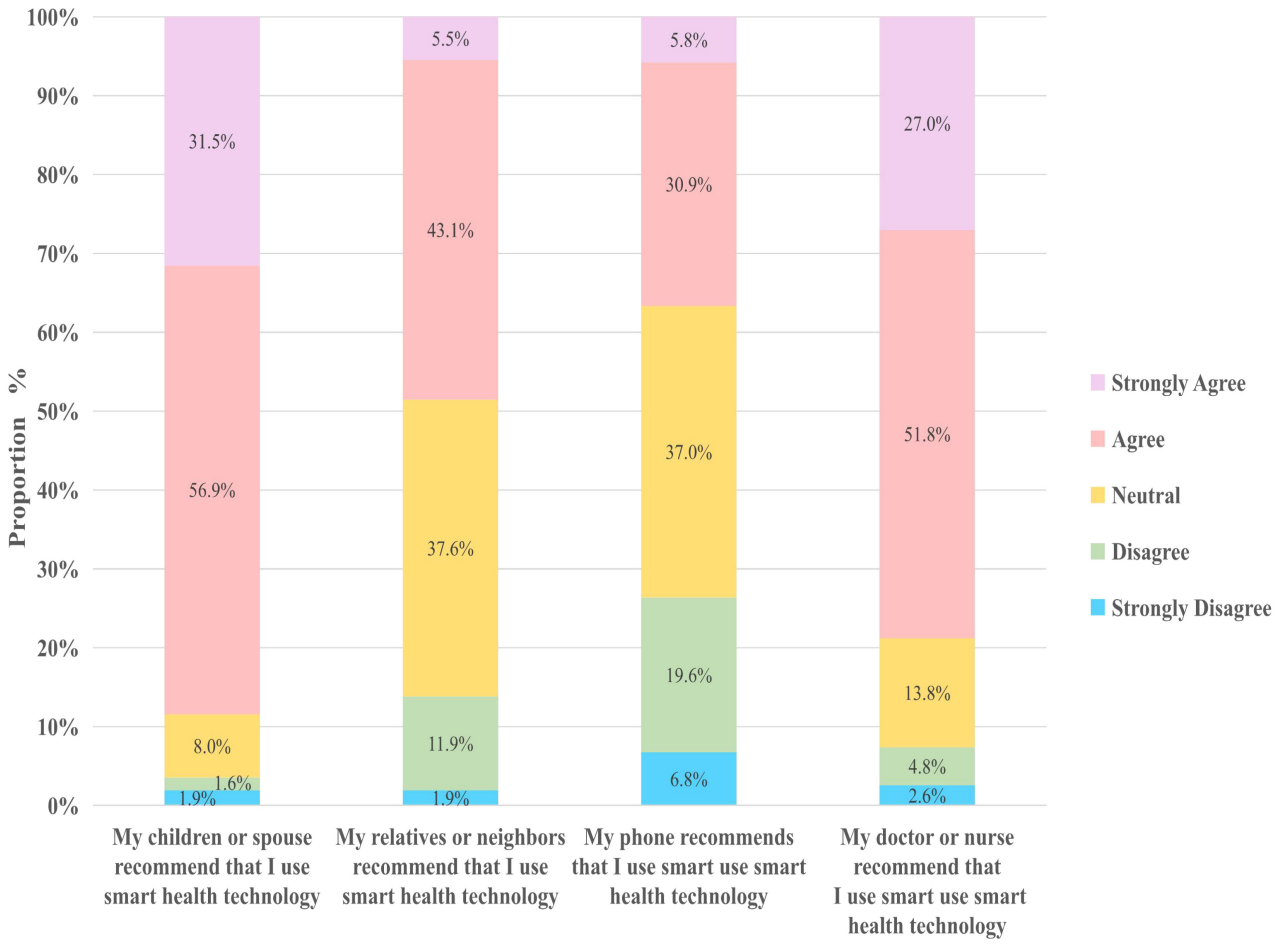
Smart health subjective norms.

Interestingly, our research also revealed that mobile phones are not the primary method by which rural older individuals access information on smart health (37%). This finding may be linked to several factors, including low smartphone usage frequency and concerns about the security and trustworthiness of mobile phones among the older rural population. More than 40% of the rural older adults in our survey expressed unfamiliarity with smart health applications on mobile phones and encountered significant challenges in operating smartphones. They also perceived smartphones as having complex functionalities and that smart health services often require digital skills, such as app usage and online payments, which further hinder their adoption (**Figure 4**).

The findings on PBC showed that 65% of the rural older adults said they possess the capability to learn and use smart health technologies. This belief is likely to be influenced by advancements in smart health technology, improvements in social promotion efforts, and other contributing factors.

In our survey, 79% of the rural older adults believed that their economic conditions allow them to use smart health technologies, a trend potentially linked to factors such as rising income levels and the lower prices of related products. Farmers’ income has increased in recent years, leading to improved economic conditions among older residents in rural areas. According to data from the National Bureau of Statistics, the average per capita disposable income of rural residents in China was 1807 CNY per month in 2023. This increase in disposable income enables them to afford basic smart health products like blood pressure monitors and oximeters. As a result, an increasing number of rural older adults can afford the costs associated with smart health, further driving their adoption of these technologies.

**Figure 5:**
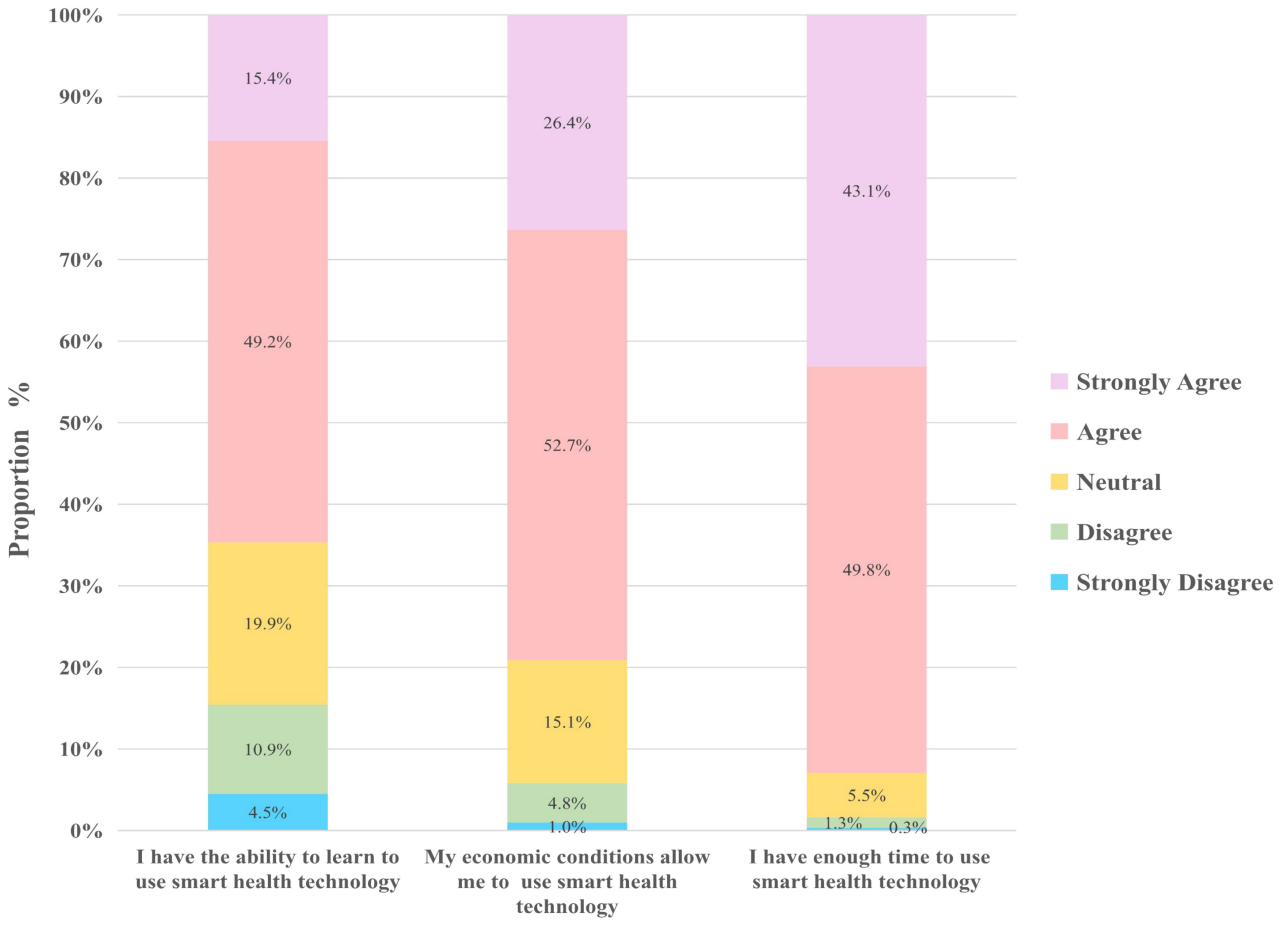
Smart health perception behavior control.

The results of our survey also showed that 93% of the older individuals in rural areas reported having sufficient time to use smart health services. This high percentage can be attributed to several factors, including improvements in rural lifestyles and convenience offered by smart health technologies. Compared with their urban counterparts, older individuals in rural areas generally experience a slower pace of life, which allows them ample time to engage in smart health services (**Figure 5**).

Smart health technologies are becoming increasingly adaptable to aging populations. Many devices and products feature larger font displays and louder voice reminders, among other design enhancements. These adjustments have effectively lowered the usability barriers for older users, making it more convenient for rural older adults to benefit from smart health services.

### 3.3 Smart health intention

When analyzing SHI, we discovered high levels of willingness among older adults in rural areas toward adopting smart health technology. Specifically, 85% expressed a readiness to use smart health technologies, 68% indicated a preference for using smart health as their primary means of health management, and 78% expressed a willingness to recommend smart health technologies to their peers. These findings underscore a strong positive inclination toward smart health solutions among rural older adults.

**Figure 6:**
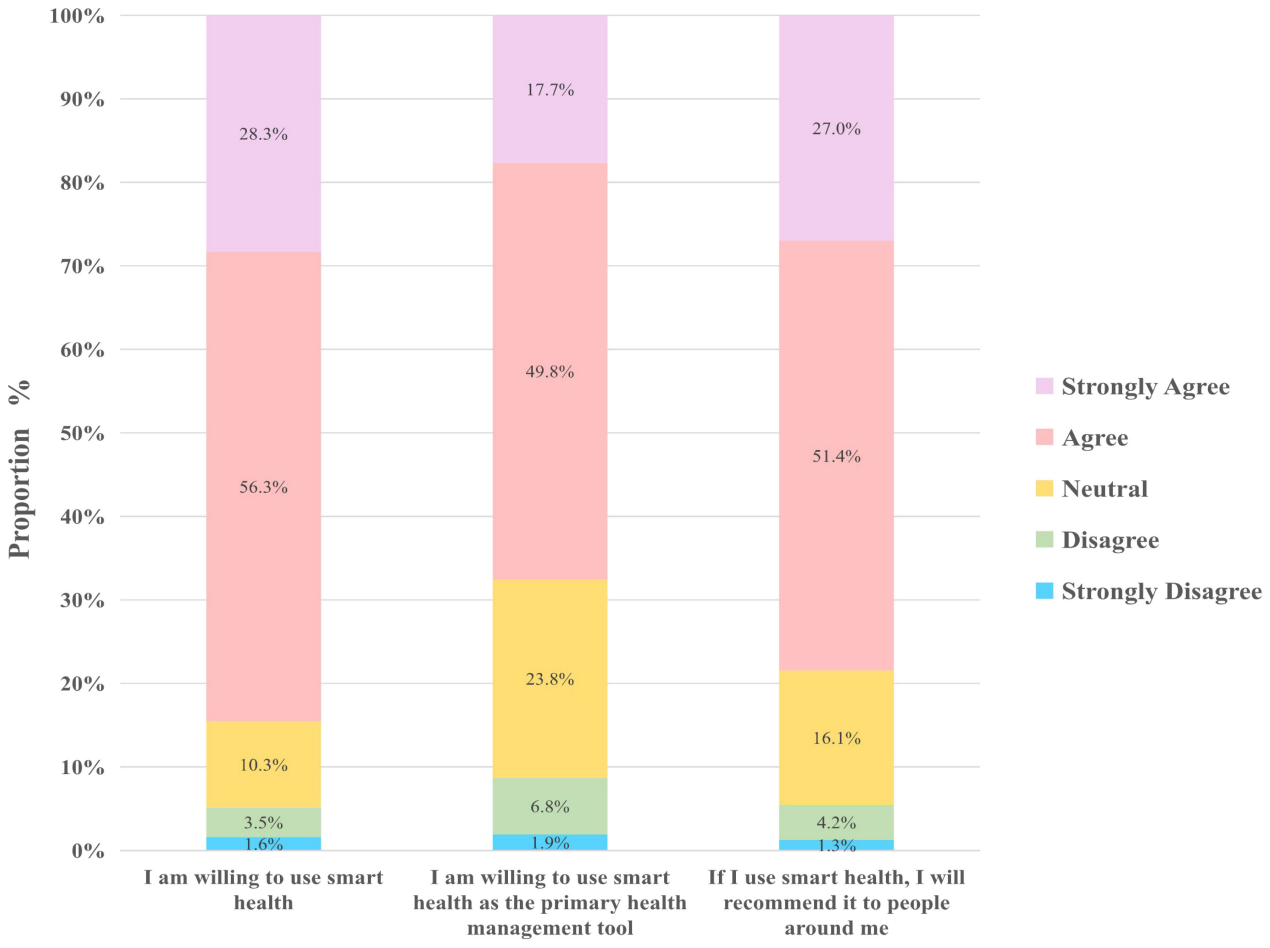
Smart health behavior intention.

Once rural older adults recognize the potential benefits of smart health, including home care convenience and enhanced health management capabilities, their willingness to adopt these technologies significantly increases. By utilizing smart health technologies, they transform their long-standing health management practices, improving self-management awareness. Additionally, as beneficiaries of smart health technologies, they recommend and influence others to adopt smart health, highlighting the potential for promoting and popularizing smart health in rural areas (**Figure 6**).

### 3.4 Structural equation modeling analysis

The analysis of structural equation modeling (SEM) enables us to assess the direct and indirect effects between variables (26). In this study, SEM was employed to investigate the relationship and influencing factors between intelligent health cognition (SHC) and intelligent health intention (SHI) among rural older participants.

#### 3.4.1 Structural equation modeling fitting results

We evaluated the model fit using Amos 20.0 software (**Table 3**). The absolute fit indices were as follows: RMSEA = 0.054, which is less than the threshold of 0.08, and GFI = 0.944, exceeding the recommended minimum of 0.9, indicating good fit. For the comparative fit indices, NFI = 0.915, TLI = 0.944, IFI = 0.957, and CFI = 0.957, all of which surpass the 0.9 threshold. Overall, each index met the recommended criteria, indicating a strong model fit. These results demonstrated that the theoretical model constructed in this study was well aligned with the behavioral intentions of rural older adults (**Table 3**).

**Table 3:** Fitting results of the smart health intention model for rural older adults.

Classification of fitting indices	Specific indicator	Recommended value	Model estimated value
Relative fit index	NFI	>0.9	0.915
	TLI	>0.9	0.944
	IFI	>0.9	0.957
	CFI	>0.9	0.957
Absolute fit index	X2/df	<2	1.917
	GFI	>0.9	0.944
	AGFI	>0.9	0.915
	RMR	<0.05	0.033
	RMSEA	<0.08	0.054
Information index	AIC	Smaller	204.196
	CAIC	Smaller	370.089

#### 3.4.2 Structural equation modeling data analysis

Based on a confirmatory factor analysis of the survey data, the critical ratio (CR) indicated that SHC and BI among rural older adults are highly significant. Specifically, the path coefficients of rural older adults’ BA and PBC on behavioral intention passed the significance level test at 1%, indicating a significant positive correlation between these factors and older adults’ use of smart health services (**Table 4**).

**Table 4:** Estimation results of the smart health willingness of rural older adults.

Observable variables	Path coefficient	Latent variables	Unstandardized Estimate	C.R.	P-value	Standardized Estimate
Behavioral intention	α21	Subjective norms	0.167	1.750	0.08	0.128
Behavioral intention	α22	Behavioral attitudes	0.991	8.902	***	0.925
Behavioral intention	α23	Perceived behavioral control	0.086	3.008	***	0.313
Subjective norms	β21	Behavioral attitudes	0.183	5.766	***	0.574
Behavioral attitudes	β22	Perceived behavioral control	0.261	5.169	***	0.635
Subjective norms	β23	Perceived behavioral control	0.172	4.779	***	0.509
BI1	r11	Behavioral intention	1	–	–	–
BI2	r12	Behavioral intention	0.999	13.704	***	0.749
BI3	r13	Behavioral intention	0.751	10.447	***	0.594
SN1	r21	Subjective norms	1	–	–	–
SN2	r22	Subjective norms	1.034	7.401	***	0.633
SN3	r23	Subjective norms	0.763	5.319	***	0.391
SN4	r24	Subjective norms	1.053	7.584	***	0.591
BA1	r31	Behavioral attitudes	1	–	–	–
BA2	r32	Behavioral attitudes	1.035	12.301	***	0.783
BA3	r33	Behavioral attitudes	1.153	12.723	***	0.809
BA4	r34	Behavioral attitudes	0.954	10.705	***	0.673
PBC1	r41	Perceived behavioral control	1	–	–	–
PBC2	r42	Perceived behavioral control	0.719	6.410	***	0.569
PBC3	r43	Perceived behavioral control	0.541	6.044	***	0.532

The three paths with “-“ indicate that they are used as the benchmark of the SEM parameter estimation in this study. *** indicates that the path coefficient passes the test at the 1% significance level.

The standardized factor loadings for “conducive to health management,” “meeting the needs of life,” and “it is necessary to promote” were 12.301, 12.723, and 10.705, respectively. These values illustrate that multiple factors influence the smart health BA of rural older adults. Particularly notable is the strong impact of “I think smart health meets my life needs” on BA, reflecting their pursuit of a healthy lifestyle and desire to enhance their quality of life. Furthermore, the path coefficient of rural older adults’ BA on behavioral intention was 0.991, indicating that BA significantly influences their intention to engage with smart health practices.

Among the SN influencing rural older adults, the standardized factor load coefficients for relatives, friends, or neighbors, mobile phones, and doctors or nurses were 7.401, 5.319, and 7.584, respectively. These values indicate that SN is influenced through different cognitive pathways. Doctors and nurses exert a strong influence on the smart health behaviors of older adults due to their trust in the professionalism of healthcare providers. This trust encourages rural older adults to adopt and practice the smart health behaviors recommended by medical professionals. With guidance from doctors and nurses, rural older adults can effectively utilize smart health technologies, such as wearable devices, for daily health monitoring and management.

The standardized factor load coefficients for “economic conditions permit” and “sufficient time” were 0.719 and 0.541, respectively, reflecting that PBC regarding smart health among rural older adults is influenced by various factors. Notably, “my economic conditions allow me to use smart health” strongly impacts the smart health behaviors of older adults, indicating that economic ability is crucial for accessing and sustaining the use of smart health products. Improved economic conditions often correlate with higher quality of life and greater health awareness. As the income of older adults increases, they tend to focus more on health management and disease prevention, embracing new health monitoring and management methods to benefit from technological advancements.

This study also identified a significant positive correlation between BA, SN, and PBC in older adults living in the rural areas of Western China. Notably, the path coefficients among these potential variables were substantial (>0.5), indicating a strong positive influence.

## 4 Discussion

This study found that over half of rural older adults in Western China (58%) are aware of smart health, indicating progress in its promotion in rural areas; however, their understanding primarily centers on wearable devices such as blood pressure monitors and oximeters. This suggests that while perceptions of smart health among rural older adults primarily focus on basic wearable devices, this recognition signals an emerging awareness of the importance of utilizing new technological tools for health monitoring and management (27).

The study reveals a significant relationship between rural older individuals’ knowledge of smart health and their readiness to engage with it. This underscores the importance of targeted cognitive interventions aimed at promoting and applying smart health. Future efforts should concentrate on enhancing rural older adults’ awareness of smart health to bolster their willingness to embrace more technologies (28).

The findings of this study underscore the challenges faced by older adults in rural Western China concerning smart health awareness and intentions. Owing to their low income and educational levels, these older individuals typically have limited opportunities to comprehend smart health technologies (29). They rely heavily on recommendations from their children, spouses, doctors, or nurses and often lack direct access to smart health solutions (30). Furthermore, current smart health technologies often do not fully address the living and health management needs specific to rural populations, specifically older adults. Issues, such as cost, technical functionalities, and relevance to the concerns of older adults, are insufficiently addressed, thereby constraining their willingness to adopt smart health solutions (31).

### 4.1 Key factors influencing older adults’ use of smart health

#### 4.1.1 Single cognition of smart health

The limited understanding of smart health among rural older adults in Western China poses a significant challenge. Although they possess a basic understanding of health monitoring devices, their overall awareness of smart health remains insufficient (32). Health management among rural older adults is influenced by their definitions of health and lifestyle habits. When faced with illness, they tend to maintain their daily routines, preferring self-treatment or selectively following medical advice rather than relying on scientifically-based clinical solutions. As the demand side of smart health, the slow acceptance of new technologies and concepts among rural older adults limits their ability to effectively comprehend and utilize smart health services, complicating efforts to introduce more advanced technologies and services. Therefore, understanding the existing knowledge of smart health among rural older adults is essential to prioritize the introduction of suitable technologies and services based on their level of awareness.

On the supply side of smart health, the government, technology companies, and medical institutions are continuously taking action. Since 2019, provinces such as Shaanxi, Sichuan, and Chongqing in western China have successively issued guidance on the development of the smart health and elderly care industry. This includes establishing smart health and elderly care pilot projects in rural areas, introducing and nurturing smart health technology companies, aiming to further promote the application of smart health among rural older adults (33). At the same time, relevant companies are continuously making improvements in the age-friendly aspects of smart health technology, constantly launching smart products with age-friendly features that cover multiple categories such as mobile phones and televisions. Clearly, we can see the series of efforts made as a smart health supplier, but have these actions enhanced the willingness of rural older adults to use such services? Therefore, it is necessary to understand the willingness of rural older adults in Western China to use smart health services and provide strategies for the reasonable supply of smart health.

The survey highlights that rural older adults rarely use mobile phones to access information about smart health (37%), suggesting the potential to increase awareness through traditional media channels such as radio and television. In addition, organizing free smart health technology demonstration events could enable older adults to experience the convenience and practicality of smart health services. This approach aims to elevate the cognitive levels of rural older adults and foster greater acceptance of smart health initiatives.

#### 4.1.2 Economic constraints

Economic constraints pose a significant challenge for rural older adults who consume smart health services. Many have limited family income and face economic hardships, with the cost of smart health services being the primary concern (79%) (34). Our survey indicates that the economic feasibility of using smart health services significantly impacts the PBC of rural older adults, highlighting their sensitivity to service costs. Despite the potential long-term health benefits and improved quality of life associated with smart health services, the initial purchase costs often deter rural older adults from adopting them.

The government can play a crucial role in addressing this issue by providing economic support to reduce the costs of smart health services. Strategies could include promoting pilot applications of smart health technologies in village clinics closer to older adult populations in rural areas. We also recommend that the government and social organizations investment more in smart health services and lower product costs through financial subsidies, tax incentives, and other measures (35).

Furthermore, providers of smart health services, including manufacturers, should focus on reducing costs and prices. Policy preferences and resource support should be directed toward the smart health industry, fostering the establishment of innovation centers, investment funds, and ecological incubators (36). This support should focus on reducing research and development (R&D) and production costs for smart health products and services.

#### 4.1.3 Technological barriers

The technological barriers to smart health technology adoption among older adults in rural areas are significant and must be addressed. Some older individuals face challenges, including age-related limitations, low education levels, and other factors, that hinder their ability to learn and use wearable health monitoring devices. Many struggle to use smartphones and other technological devices, and find it difficult to understand the complex operation processes and interpret the data (37). These barriers not only impede older adults’ acceptance of smart health technologies and services, but also diminish their long-term willingness to utilize them.

To address these challenges, technology enterprises should enhance product adaptability for aging populations by simplifying the application interfaces and operational processes for smart health products to enhance the user experience. Specifically, smart health manufacturers should consider the habits and needs of older users when designing products by employing straightforward initialization interfaces, guided operation processes, high-volume voice prompts, and one-click help functions to reduce usability challenges (38).

#### 4.1.4 Lack of Social and Family Support

The lack of social and family support poses a significant challenge to the adoption of smart health technologies in underdeveloped rural areas, particularly in Western China.

On the one hand, family structures in rural communities foster a strong sense of trust among older adults toward their family members, making them more receptive to advice and information from children or spouses. These trusted family members serve as primary channels for gathering information. Owing to close-knit family structures and limited social support systems, many older adults lack the necessary assistance and guidance to utilize smart health services independently. In the absence of close family members, they may struggle to access and benefit from smart health services in a timely manner, reducing their willingness to adopt these technologies (39).

On the other hand, doctors and nurses, as professionals with medical expertise, possess significant authority and credibility in treatment and health management. Their professional authority contributes significantly to building trust among older adults in rural areas.

To address this issue, efforts should focus on enhancing social and family support networks for smart health initiatives, enabling rural older individuals to benefit effectively from lifestyle improvements facilitated by smart health services. First, enhancing smart health service capabilities in village clinics and providing local support initiatives can ensure that rural older adults receive the necessary assistance and guidance during the initial phases of smart health adoption (40). Second, family members, including children and spouses, should be encouraged to actively participate in the health management of older relatives in rural areas, thereby promoting the widespread adoption and utilization of smart health services (41). Adequate family support is crucial for overcoming the practical challenges and psychological barriers that older adults face when understanding and using smart health technologies (42).

## 5 Conclusion

Our findings have significant implications for the promotion and implementation of smart health technologies in less developed areas. Our results reveal that rural older individuals in Western China possess a basic understanding of smart health and have a pressing demand for health management and medical services. Although their understanding may not be comprehensive, and they tend to focus on specific types of smart health technologies, this signifies notable progress in the promotion of smart health in rural areas. Once the older adults in our study grasped the concept, potential benefits, technical products, and application scenarios of smart health, most expressed a belief that smart health would facilitate effective health management and meet their life needs, underscoring the necessity of its promotion in rural settings.

Regarding approaches to understanding smart health, the older adults showed a preference for recommendations from their children or spouses, followed by guidance from healthcare professionals such as doctors or nurses. Generally, rural older adults in Western China exhibited a strong willingness to engage in smart health initiatives. After using smart health technologies, they were inclined to adopt them as their primary method of health management and were willing to recommend smart health technologies to their peers, presenting promising prospects for the widespread adoption and application of smart health in underdeveloped regions.

In addition, our study established a link between smart health cognition and behavioral intention among older adults living in rural Western China. We found that PBC and behavioral attitudes toward smart health directly influenced older adults’ intention to use these technologies. Factors, such as meeting life needs, recommendations from doctors or nurses, and economic conditions, were identified as the primary drivers shaping older adults’ engagement with smart health technologies.

Based on these insights, we recommend several strategies to promote the adoption and application of smart health among rural older adults in Western China. Strategies include enhancing public awareness and education about smart health through traditional media channels, such as radio and television, reducing the cost of smart health technologies and services, simplifying application interfaces and operations, and strengthening societal support, particularly from family members. Overall, our study offers valuable insights that can guide efforts to promote healthy aging and enhance medical strategies for underdeveloped regions by elucidating the smart health cognition and behavioral intentions of rural older adults in Western China.

## 6 Limitations

This study has several limitations that warrant acknowledgment. The primary limitation is that the online questionnaire completed by children on behalf of their parents may not fully reflect the cognition and intentions of older adults regarding smart health. Given the generally lower education levels among rural older adults in Western China (75% having a secondary school education or below), their usage rates of smartphones and computers are low. Consequently, obtaining authentic data from them through online questionnaires is challenging. To address this, we relied on their children to respond on their behalf. Future studies will aim to utilize face-to-face interviews to obtain more accurate information about rural older adults. Although 88% of participants lived with their parents, this still affects the authenticity of the survey results, introducing potential risks of information bias and subjective interpretation.

Additionally, this study was conducted within a specific geographical area, which may limit the generalizability of the findings. Given that the sample consisted solely of elderly individuals from rural areas in Western China, further research is necessary to validate the applicability of our results across diverse cultural contexts.

Despite these limitations, our research provides valuable insights into the smart health cognition and intentions of elderly populations in rural Western China.

## Data Availability

The raw data supporting the conclusions of this article will be made available by the authors, without undue reservation.
